# Non-typhoidal *Salmonella *bacteraemia: Epidemiology, clinical characteristics and its' association with severe immunosuppression

**DOI:** 10.1186/1476-0711-8-15

**Published:** 2009-05-18

**Authors:** Amreeta Dhanoa, Quek Kia Fatt

**Affiliations:** 1 School of Medicine and Health Sciences, Monash University, Sunway Campus, Lagoon Selatan Road, Sunway, Malaysia

## Abstract

**Background:**

Non-typhoidal *Salmonella *(NTS) is increasingly recognized as an important pathogen associated with bacteraemia especially in immunosuppressed patients. However, there is limited data specifically describing the clinical characteristics and outcome amongst the immunosuppressed patients.

**Methods:**

A total of 56,707 blood culture samples and 5,450 stool samples were received by the microbiology laboratory at a tertiary referral hospital in Malaysia, during a 4-year study period. Out of these samples, 55 non-duplicate NTS isolates were identified from blood and 121 from stool. A retrospective analysis of the 55 patients with NTS bacteraemia was then conducted to determine the predominant NTS serovars causing bacteraemia and its' blood invasive potential, epidemiological data, clinical characteristics and antimicrobial susceptibility. Patients were then grouped as immunosuppressed and non-immunosuppressed to determine the association of severe immunosuppression on clinical features. Data was analyzed using the Statistical Package for Social Sciences (SPSS version 15.0) using the non-parametric Mann-Whitney test, Fisher's exact test or Chi-squared test. The odds ratio (OR) and its 95% confidence intervals (CI) were calculated. The *P*-value < 0.05 (two-tailed) was taken as the level of significance.

**Results:**

Out of 55 NTS bacteraemia cases identified, 81.8% (45/55) were community-acquired. *Salmonella enterica *serovar Enteritidis had the highest blood invasiveness. An extra-intestinal focus of infection was noted in 30.9% (17/55) of the patients, most commonly involving the lungs and soft tissue. 90.9% (50/55) of the patients had an underlying disease and 65.5% (36/55) of the patients had severe clinical immunosuppressive condition with malignancy and HIV being the most common. Immunosuppressed patients had higher mortality (P = 0.04), presented more commonly with primary bacteraemia (P = 0.023), leukopenia (P = 0.001) and opportunistic infections (P = 0.01). In contrast, atherosclerotic conditions (P = 0.015), mycotic aneurysms (0.037) and gastroenteritis (P = 0.03), were significantly more common in the non-immunosuppressed patients. The non-immunosuppressed group also had a higher proportion of older patients (>50 years) with a significantly higher median age (64 versus 36.5 years; p = 0.005).

**Conclusion:**

Patients with severe clinical immunosuppression had higher mortality, presented more commonly with primary bacteraemia, leukopenia and opportunistic infections and absence of gastroenteritis. Early identification and prompt medical treatment can be life saving because of the high mortality and morbidity associated with this disease especially in the immunosuppressed patients.

## Background

Non-typhoidal *Salmonella *(NTS) species are important food borne pathogens with acute gastroenteritis being the most common clinical manifestation. However, invasion beyond the gastrointestinal tract occurs in approximately 5% of the patients with NTS gastroenteritis resulting in bacteraemia [1]. Certain serovars of *Salmonella *show a much higher predilection for causing bacteraemia and these serovars differ in different countries [2,3]. As a larger proportion of patients have become immunocompromised either due to certain primary diseases or modern aggressive therapies, the occurrence of NTS invasive disease appears to be increasing [4]. Although NTS infections have been widely studied in medical literature, few studies have focused specifically on NTS bacteraemia. Moreover, data specifically describing the epidemiological and clinical characteristics amongst the immunosuppressed patients is scarce.

Therefore, this study was undertaken to determine the a) blood invasive potential and the predominant NTS serovars causing bacteraemia in a tertiary hospital in Malaysia b) clinical characteristics and risk factors associated with NTS bacteraemia c) effects of severe immunosuppression on clinical presentation and outcome.

## Methods

### Study design and selection of patients

A retrospective study was performed from July 2002 till July 2006 involving patients admitted at Penang General Hospital, a 1100-bedded Malaysian hospital that functions as a tertiary referral hospital for many disciplines including haematology, oncology, rheumatology and infectious diseases as well as serving the local population. A total of 56,707 blood samples and 5,450 stool samples were received and processed at the microbiology laboratory during the study period. The clinical notes of the 55 patients identified to have NTS bacteraemia were reviewed retrospectively. For patients with recurrent NTS isolated from blood and stool, only the first episode was included for analysis. The following data was obtained from patients with NTS bacteraemia: demographic characteristics, clinical manifestations, possible predisposing factors, antibiotic susceptibilities, treatment and clinical outcome. Relevant data from the 121 non-duplicate NTS isolated from stool was obtained from the microbiological records to determine the 'blood invasiveness ratio' (BIR).

### Case definition

BIR reflects the blood invasion potential of a particular NTS serovar and is calculated as the ratio of blood isolates (B) to the total number of blood and stool isolates (T) for each *Salmonella *serovar (B/T ratio) and expressed per 100 blood+stool isolates [5]. An extra-intestinal focus of infection (EFI) was defined as the presence of clinical or radiological evidence of infection supported by isolation of NTS from a site other than stool specimen. Bacteraemia was classified as primary or secondary according to the classification proposed by Ramos JM et al. [6], with some modification. Primary bacteraemia was diagnosed when NTS was isolated from one or more blood cultures with no isolation from other infected foci and no history of recent gastroenteritis. The term secondary bacteraemia was used when isolation of NTS from blood followed an episode of gastroenteritis and if *Salmonella *was not isolated from any source except stool.

Community-acquired bacteraemia was defined as bacteraemia that develops in a patient prior to admission, or a positive blood culture obtained within 48 hours of hospital admission, in patients without history of hospitalization in the 2 weeks prior to the current bacteraemic episode. The infection was considered hospital-acquired if it developed 48 hours or later after admission.

Paediatric population was defined as patients aged 16 years or less. Patients were considered to have severe clinical immunosuppression when any of the following conditions were present; human immunodeficiency virus (HIV) infection, use of immunosuppressive therapy, neutropenia (absolute neutrophil count < 500/mm^3^), malignancy or autoimmune disease. Immunosuppressive therapy included recipients of corticosteroids (at least 10 mg of prednisolone/day or equivalent corticosteroids) or chemotherapy for malignancy received within 2 weeks prior to bacteraemia. Atherosclerotic conditions were defined as having atherosclerotic diseases or risk factors for atherosclerosis (previous ischaemic stroke, ischaemic heart disease, diabetes mellitus and hypertension).

Shock was defined as systolic blood pressure ≤ 90 mm Hg or an unstable hemodynamic status requiring inotropic agents to maintain blood pressure and was considered bacteraemia related if there was no other obvious reason for it. Renal dysfunction was defined as a twofold increase in baseline serum creatinine level or an increase in serum creatinine level of 176.8 μmol/L (2.0 mg/dL), leukopenia (<4 000 leukocytes/mm^3^) and leukocytosis (>11 000 leukocytes/mm^3^). Antibiotic treatment was deemed adequate if at least one antibiotic used within the first 72 hours of a positive blood culture had in-vitro activity against the *Salmonella *strain isolated. Antibiotics considered potentially active included ampicillin and its' derivatives (ampicillin-sulbactam, amoxicillin/clavulanate), chloramphenicol, ceftriaxone, ciprofloxacin and cotrimoxazole.

### Microbiology

All blood cultures were processed using the Bactec 9240 fluorescent system (Becton Dickinson). All isolates were identified as *Salmonella *according to standard microbiological techniques [7]. Serotyping of all *Salmonella *isolates was performed according to Kaufmann-White scheme using O and H antisera (Difco) [8] either at the local microbiology laboratory or at the regional public health laboratory. Other laboratory data included antibiotic susceptibilities, time taken for BACTEC culture bottles to turn positive and some haematology parameters. Antibiotic susceptibility was determined by means of Kirby-Bauer disk diffusion method using the guidelines provided by Clinical Laboratory Standard Institute (CLSI), formerly known as National Committee for Clinical Laboratory Standards (NCCLS) guidelines [9]. The antimicrobial agents routinely tested included ampicillin, chloramphenicol, trimethoprim-sulfamethoxazole, ciprofloxacin and ceftriaxone.

### Statistical analysis

Clinical data of all patients was obtained until discharge or death. Statistical analysis was then performed to determine the association of severe immunosuppression on clinical and epidemiological characteristics. Data was analyzed using the Statistical Package for Social Sciences (SPSS version 15.0). Continuous variables were expressed as median ± interquartile range (IQR) and comparison was made using the non-parametric Mann-Whitney test. Categorical variables were expressed as numbers and percentages and comparison amongst variables was determined by the Fisher's exact test or Chi-squared test. The odds ratio (OR) and its 95% confidence intervals (CI) were calculated. The *P*-value < 0.05 (two-tailed) was taken as the level of significance.

## Results

### Serogroup and serovar distribution of NTS

A total number of 56,707 blood samples were received by the microbiology laboratory for bacterial culture during the study period, from which 6,015 (10.6%) samples were positive for bacterial growth. Among the positive blood samples, 55 non-duplicate NTS isolates were identified. Similarly, a total number of 5,450 stool samples were received for bacterial culture during this period, from which significant stool pathogens were identified in 317 samples (5.8%). Non-duplicate NTS isolates were cultured from 121 patients. The distribution of these NTS isolates according to serovars and the 'blood invasiveness ratio' (BIR) is presented in Table 1.

**Table 1:** Serovar distribution of NTS in blood and stool and its' blood invasive ratio (BIR)

Serovar	Serogroup	Blood isolates (%) n = 55		Stool isolates (%) n = 121		BIR*
Enteritidis	D	40	(72.7)	19	(15.7)	67.8
Paratyphi b	B	3	(5.5)	8	(6.6)	27.3
Choleraesuis	C	1	(1.8)	3	(2.5)	25.0
Senflenberg	E	1	(1.8)	3	(2.5)	25.0
Blegdam	D	3	(5.5)	10	(8.3)	23.0
Tshiongwe	C	2	(3.6)	8	(6.6)	20.0
Hato	B	1	(1.8)	4	(3.3)	20.0
Corvallis	C	4	(7.2)	23	(19.0)	14.8
Waltevreden	E	0	(0.0)	14	(11.6)	0
Limete	B	0	(0.0)	8	(6.6)	0
Typhimurium	B	0	(0.0)	6	(5.0)	0
Others		0	(0.0)	15	(12.4)	0

* BIR = (B/T) × 100 (B = blood isolates and T = total blood+stool isolates)

### Demographic features

Male patients predominated (67.3%). No age group was excluded, with the youngest patient being one year and 4 months and the oldest 89 years (median ± IQR, 39 ± 43 years). There were thirteen (23.6%) paediatric patients. Majority of the patients were Malays (49%) followed by Chinese (42%), Indians (7%) and others (2%).

### Clinical characteristics and outcome

Fifty patients (90.9%) had at least one underlying disease predisposing them to NTS bacteraemia. The predisposing factors are illustrated in Table 2. In four patients, more than one risk factors were identified; 2 patients with SLE had renal failure requiring hemodialysis, 1 patient with AIDS had liver cirrhosis due to hepatitis B and 1 patient with carcinoma of pancreas had underlying ischaemic heart disease.

**Table 2:** Clinical characteristics of patients with NTS bacteraemia

Characteristics	No of cases (n = 55)	%
**Underlying condition**		
Severe immunosuppressive conditions *	36	65.5
Malignancy	13	23.6
Solid†	9	
Haematological‡	4	
AIDS	11	20.0
SLE	6	10.9
Hypogammmaglobulinaemia	2	3.6
Immunosuppressives	15	27.3
Atherosclerotic conditions *	8	14.5
Hypertension	3	5.5
Diabetes mellitus	4	7.3
Ischaemic heart disease	2	3.6
Stroke	2	3.6
Liver cirrhosis§	4	7.3
Renal disease	4	7.3
Others^¶^	2	3.6
**Sites of infection**		
Blood only	38	69.1
EFI**	17	30.9
Lung	4	7.3
Soft tissue	4	7.3
Bone and joint	3	5.5
Meningitis	3	5.5
Mycotic aneurysm	3	5.5
Urinary tract	2	3.6
Peritonitis	1	1.8

* A patient may have more than one conditions under these categories (severe immunosuppressive or atherosclerotic categories), therefore the total number of conditions may not tally with the main category

**A patient may have more than one EFIs

†Includes carcinoma rectum, pancreas, stomach, colon, liver, brain glioma, yolk sac tumor of lung, osteosarcoma, lymphoma

‡ Includes acute lymphoblastic leukaemia (n = 3), acute myeloid leukaemia (n = 1)

§Includes Hepatitis B (n = 2) alcoholic liver cirrhosis (n = 1), Hepatitis C (n = 1)

¶Includes travel from Pakistan (n = 1), Thalassaemia (n = 1)

Thirty-six patients (65.5%) had at least one severe clinical immunosuppressive condition. Underlying malignancy (23.6%) was the most common risk factor followed by AIDS/HIV (20%). Eight patients with underlying malignancy and 3 patients with SLE were on immunosuppressives. Majority of the cases (45/55, 81.8%) presented with community-acquired bacteraemia. In one-fifth of the cases, bacteraemia was hospital-acquired with a detection time of 8 to 30 days after admission.

Seventeen patients (30.9%) had at least one extra-intestinal focus of infection (EFI). In three cases, more than one organ was involved. Lung and soft tissue were the most common extra-intestinal sites involved. All the four soft tissue infections had microbiological evidence of infection either from pus or tissue; two had soft tissue abscesses (chest wall and pararenal), one had an infected bedsore and one case had a highly unusual presentation of necrotizing fasciitis. Pneumonia was associated with isolation of NTS from respiratory secretions and in one case it was complicated by empyema, requiring chest tube drainage. Of the three cases involving bone and joint, two had septic arthritis and one presented with an infected knee implant. Three patients developed radiologically confirmed aortic mycotic aneurysm, out of which one case was fatal. All three mycotic aneurysms occurred in patients above 65 years of age and each had at least one atherosclerotic condition, (diabetes mellitus in two patients and ischaemic heart disease in one). Meningitis was a feature in two children aged 2 and 3 years and a 39 years old male. NTS was isolated from cerebrospinal fluid from all 3 cases. All three cases of meningitis had an underlying risk factor (agammaglobulinaemia, thalassaemia and AIDS respectively) and one case was complicated by subdural effusion. Bacteraemia was associated with NTS urinary tract infection in two cases, both occurring in males aged > 70 years with an associated benign prostatic hyperplasia. Thirty-eight patients (69%) had bacteraemia without any obvious EFIs.

Thirty-four patients (61.8%) received initial empiric antibiotic therapy that was appropriate for treatment of NTS bacteraemia in the first 72 hours. Of these ampicillin and its' derivatives (ampicillin/sulbactam and amoxicillin/clavulanic) were administered to 11 patients; ceftriaxone (17); ciprofloxacin (3); cefotaxime (2) and cotrimoxazole (1).

The overall mortality rate was 21.8% (12 of 55). Among patients who survived, the median (± IQR) duration of hospitalization was 9 days (± 13).

### Antibiotic susceptibility

The antibiotic resistance rates was highest with ampicillin (30.9%), followed by trimethoprim-sulfamethoxazole (18.2%). Ceftriaxone, ciprofloxacin and chloramphenicol were fully susceptible in all the isolates.

### Association of severe immunosuppression on epidemiological and clinical characteristics

Table 3 shows the comparison between patients with or without severe immunosuppressive conditions. Primary bacteraemia was significantly more common as a presenting feature in the immunosupressed patients compared to the non-immunosuppressed. The immunosuppressed group also had significantly higher mortality rates (11/36, 30.6%). Opportunistic infections were associated only with the severely immunosuppressed group. Eight of the eleven patients with HIV had concomitant opportunistic infections (six had *Mycobacterium tuberculosis *and one each had *Penicillium marneffei *and *Toxoplasma gondii*). Candidaemia was present in two patients with underlying malignancy and one patient with SLE manifested with tuberculosis.

**Table 3:** Comparison between NTS bacteraemia patients with or without severe immunosuppression

Variable	Overall n(%)/median(± IQR) (n = 55)	Severe immunosuppression n(%)/median (± IQR)		p	OR	95%C1
		Yes (n = 36)	No(n = 19)			
Age > 50 ^a^	18(32.7)	6(16.7)	12(63.2)	0.001	0.12	0.03–0.42
Male ^b^	37(67.3)	24(66.7)	13(68.4)	0.89	0.92	0.28–3.034
EF1 ^b^	17(30.9)	10(27.8)	7(36.8)	0.49	0.66	0.20–2.15
Mycotic aneurysm ^a^	3(5.5)	0(0)	3(15.8)	0.037	undefined	
Gastroenteritis ^b^	16(29.1)	7(19.4)	9(47.4)	0.03	0.27	0.079–0.91
Primary bacteraemia ^b^	23(41.8)	19(52.8)	4 (21.1)	0.023	4.19	1.16–15.11
Atherosclerosis ^a^	12(21.8)	4(11.1)	8(42.1)	0.015	0.17	0.043–0.69
Mortality^a^	12(21.8)	11(30.6)	1(5.3)	0.041	7.92	0.94–66.97
*Salmonella *Enteritidis ^b^	40(72.7)	29(80.6)	11(57.9)	0.073	3.013	0.88–10.30
Group D *Salmonella*^a^	43(78.2)	31(86.1)	12(63.2)	0.084	3.62	0.96–13.64
Hospital acquisition ^a^	10(18.2)	9(25.0)	1 (5.3)	0.14	6	0.70–51.53
Septic shock ^a^	5(9.1)	4(11.1)	1(5.3)	0.65	2.25	0.23–21.69
Fever ^a^	42(76.4)	27(75)	15(78.9)	1.00	1.25	0.33–4.76
Paediatric ^a^	13(23.6)	10(27.8)	3(15.8)	0.51	0.49	0.12–2.04
Opportunistic infections ^a^	11(20)	11(30.6)	0(0)	0.01	Undefined	
Leukopenia ^b^	15(27.3)	15(41.6)	0(0)	0.001	undefined	
Blood culture positivity < 24 hours ^b^	35(63.6)	24(66.7)	11(57.9)	0.52	1.45	0.46–4.57
Age (years) ^c^	39(43.0)	36.5(31.25)	64.0(41.0)	0.005	NA	
Duration of hospitalization (days) ^c^	9(13.0)	8.0(16.0)	10.0(12.0)	0.18	NA	
Leukocytes/mm^3^^c^	7.7(7.0)	5.5(7.18)	8.3(5.7)	0.016	NA	
Lymphocytes/mm^3 ^^c^	1.1(1.4)	0.9(1.0)	1.9(2.2)	0.026	NA	
Duration of symptoms (days) ^c^	5(4.0)	5.0(4.0)	5.0(4.0)	0.98	NA	

^a ^Fisher's exact test; ^b ^Chi-squared test; ^c ^Non-parametric Mann-Whitney test.

^a ^and ^b ^expressed as number (%); ^c ^expressed as median (± IQR)

NA = not applicable

Overall, fifteen patients presented with leukopenia (27.3%) and thirteen with leukocytosis (23.6%). Patients with severe immunosuppression had a higher proportion of leukopenia and significantly lower leukocyte and lymphocyte counts. Hospital acquisition of bacteraemia and bacteraemia caused by *Salmonella enterica *serovar Enteritidis and Group D *Salmonella *was notably higher in the immunosuppressed patients although it was not statistically significant. In contrast, gastroenteritis, atherosclerotic conditions and mycotic aneurysm were significantly more common in the non-immunosuppressed patients. The non-immunosuppressed group also had a higher proportion of older patients (>50 years) with a significantly higher median age (64 versus 36.5 years; p = 0.005). The median length of hospitalization was longer in the non-immunosuppressed group (10 days versus 8 days) possibly related to increased early mortality in the severely immunosuppressed group, although it was statistically not significant.

## Discussion

NTS is increasingly recognized as an important pathogen associated with bacteraemia in both the immunosuppressed as well as the immunocompetent patients. In the present study, serogroup D had the highest blood invasiveness with 40 out of the 43 (93%) serogroup D isolates belonging to *Salmonella enterica *serovar Enteritidis. *Salmonella enterica *serovar Typhimurium [3], the most common serotype isolated in Malawi was not noted in any of our bacteraemic patients. Taiwan demonstrates a high prevalence of serogroup B and C [10] and Thailand has a higher proportion of serogroup C [2] amongst their bacteraemic patients. The knowledge about the prevalent serotypes with high invasive potential is of epidemiological and public health importance.

The extra-intestinal focus of infection (EFI) rate of 30.9% in our study was higher than that reported by Galofre J et al. [11]. However, EFI rates of as high as 47% have been reported in Taiwan involving patients with *Salmonella enterica *serovar Choleraesuis bacteraemia [12]. Contrary to previous studies which found higher percentage mycotic aneurysms amongst their NTS bacteraemic patients [12,13], our study had significantly lower rates; an overall rate of 5.5% which increased to 17% amongst patients > 50 years (P = 0.031). The risk factors for vascular infection in NTS bacteraemic patients which includes age > 50 and underlying atherosclerosis [12,14] were present in our patients and this serious complication should always be kept in mind when an elderly patient presents with NTS bacteraemia. The overall low rates of mycotic aneurysm in the present study could be partly attributed to very low prevalence of *Salmonella enterica *serovar Choleraesuis bacteraemia, which has a high predilection for causing bacteraemia and mycotic aneurysm [12,13]. The presence of mycotic aneurysm and atherosclerotic conditions were significantly lower in the immunosuppressed patients in this study (p < 0.05). Although immunosuppression predisposes patients to bacteraemia, it has a contradictory role in the pathogenesis of endovascular infection [14], suggesting the possible role of immune system in the process of inflammation and destruction of aorta and heart valves.

In recent years, NTS bacteraemia has been increasingly reported as a cause of life-threatening infection in immunocompromised hosts. It is worth noting that, in our study, malignancy and HIV/AIDS were the two most common conditions associated with NTS bacteraemia. As suggested by several recent reports in Thailand and Malawi, NTS bacteraemia has become increasingly important amongst patients with HIV [2,15]. Multiple series have identified malignancy as a risk factor for NTS bacteraemia [14]. SLE was the predisposing disease in 10.9% of our cases. In a recent series involving SLE bacteraemic patients [16], NTS was the most common gram-negative bacteria isolated. In the present study, significantly lower proportion of patients >50 years had severe immunosuppressive conditions like HIV and malignancy, instead they presented with age-related conditions like atherosclerosis.

The findings in the present study showed that the presence of primary bacteraemia and absence of gastroenteritis was more common in patients with severe immunosuppression and this concurred with the findings of Ramos et al. [17]. Underlying immunosuppression should be excluded in patients presenting with NTS bacteraemia in the absence of gastroenteritis [17,18].

Although the overall mortality in the present study was 21.8% (12/55), 11 of the 12 deaths actually occurred in patients with severe immunosuppressive diseases. Therefore, in the present study the deaths were most likely attributable to the underlying conditions. Variations in mortality rates associated with NTS bacteraemia ranging from 12% [11] to 47% [15] has been reported in previous studies. These wide ranges may reflect the differences in the severity of underlying diseases, the serovars of NTS, the availability of antimicrobials and the antimicrobial resistance of NTS in various countries.

A predisposition to NTS bacteraemia in immunosuppressed patients and a trend towards higher mortality observed in patients with immunosuppressed conditions illustrate the importance of immune system in defense against *Salmonella *infection. Cell mediated immunity is vital for defense against intracellular bacteria like *Salmonella *and leukocytes especially lymphocytes are essential host defense. In the present study, severely immunosuppressed patients presented with significantly lower lymphocyte counts. Cytokine deficiencies such as interleukin IL-12/IL-23 and interferon gamma can predispose one to invasive NTS infections [4,19]. Equally important is to determine the virulence of *Salmonella *strains which may favor systemic dissemination in the host. Direct inhibition of T lymphocyte activation, the interference of professional antigen presenting cells function to process and present *Salmonella*-expressed antigens to T cell are some of the immune evasion techniques to escape immune detection by virulent *Salmonella*. The elucidation of these virulent factors will allow for the design of new strategies to prevent the systemic diseases caused by this pathogen [20].

A major limitation of the present study was that it was based in a tertiary hospital and hence might be biased towards patients with invasive NTS diseases. Moreover, most patients attended local health clinics for diarrhoeal diseases. Therefore, our data on blood invasiveness ratio which is based on positive stool and blood isolates obtained from the microbiology laboratory may not be reflective of the true incidence. However, it provides a relative comparison between the different serovars. Secondly, owing to the retrospective nature of the study, the presence of an extra-intestinal focus of infection could not be verified for all patients. Thirdly, since our study was conducted in a tertiary hospital where a high proportion of patients are immunosupressed as a result of underlying disease or modern aggressive therapy, similar results may not be extrapolated to other institutions in Malaysia where the patient population might differ.

## Conclusion

In conclusion, serogroup D *Salmonella *had the highest blood invasiveness and almost all these isolates belonged to serotype *Salmonella enterica *serovar Enteritidis. Two-third of patients had at least one severe immunosuppressive condition with malignancy and HIV being the most common. An extra-intestinal focus of infection was noted in one third of patients, most commonly involving the lung and soft tissue. Patients with severe clinical immunosuppression had higher mortality, presented more commonly with primary bacteraemia, leukopenia and opportunistic infections and absence of gastroenteritis. Early identification and prompt medical treatment can be life saving because of the high mortality and morbidity associated with this disease especially in the immunosuppressed patients.

## Ethical approval

Ethical approval to review the data was obtained from Clinical Research Centre, Penang General Hospital

## Competing interests

The authors declare that they have no competing interests.

## Authors' contributions

AD designed the study, collected, analyzed and interpreted the data and wrote the manuscript. QKF assisted in analyzing the data and reviewed the manuscript.
